# Spray-cast multilayer perovskite solar cells with an active-area of 1.5 cm^2^

**DOI:** 10.1038/s41598-017-08642-2

**Published:** 2017-08-11

**Authors:** James E. Bishop, David K. Mohamad, Michael Wong-Stringer, Alex Smith, David G. Lidzey

**Affiliations:** 1 Department of Physics & Astronomy, University of Sheffield, Hicks Building, Hounsfield Road, Sheffield, S3 7RH UK; 2 CREST, Wolfson School, Loughborough University, Loughborough, Leicestershire LE11 3TU UK

## Abstract

We utilise spray-coating under ambient conditions to sequentially deposit compact-TiO_2_, mesoporous-TiO_2_, CH_3_NH_3_PbI_(3−x)_Cl_x_ perovskite and doped spiro-OMeTAD layers, creating a mesoporous standard architecture perovskite solar cell (PSC). The devices created had an average power conversion efficiency (PCE) of 9.2% and a peak PCE of 10.2%; values that compare favourably with control-devices fabricated by spin-casting that had an average efficiency of 11.4%. We show that our process can be used to create devices having an active-area of 1.5 cm^2^ having an independently verified efficiency of 6.6%. This work demonstrates the versatility of spray-coating as well as its potential as a method of manufacturing low-cost, large-area, efficient perovskite devices.

## Introduction

Within the last seven years, devices based on perovskite absorbers have emerged as a leading thin-film photovoltaic (PV) technology, having power conversion efficiencies (PCEs) rising from 3.8%^1^ to over 20%^2^. Perovskites combine the semiconducting properties typically associated with inorganic photovoltaics, such as strong light absorption^3, 4^, high charge-carrier mobility^4, 5^, tuneable bandgap^6, 7^ and low recombination rates^8, 9^ with ease of processing from solution. As a result perovskite based photovoltaics are predicted to have a shorter energy payback time than current commercial technologies of less than half a year^10^.

Spin-coating remains the principal method for thin-film preparation in high performance perovskite solar cells (PSCs)^11^. Whilst this method is capable of delivering films of well-defined thickness and high uniformity, it is inherently unsuitable for large-scale PSC manufacture. If PSCs are to fulfil their promise as a low-cost, high-volume source of sustainable energy, their deposition must be achieved using truly scalable techniques^12^. This is a growing area of research, with perovskite materials now being deposited by ink-jet printing^13^, slot-die coating^14^, doctor-blading^15^ and spray-coating^16–19^.

Spray-coating is a versatile coating technique that is widely employed in industry. It can be used to deposit functional films at high coating-rates, over large areas^20, 21^ with high material utilisation^22^. It also has the ability to apply conformal coatings to irregular surfaces^23, 24^. Spray-coating has already been applied to the fabrication of standard architecture planar PSCs *via* single-step spray-deposition of MAPbI_3−x_Cl_x_^19^ and MAPbI_3_^25^ reaching a PCE of up to 13%. By introducing PbAc_2_ into the precursor ink, Tait *et al*.^17^ demonstrated that such devices could reach a PCE of 15.7% though the development of a dense, highly uniform perovskite crystal lattice. Comparable device performance has been demonstrated by Huang *et al*.^18^ through the development of a two-step spray-cast MAPbI_3_ perovskite deposition protocol in which a thin-film of PbI_2_ was first spray-cast onto mesoporous TiO_2_. Onto this was spray-cast a film of methyl-ammonium iodide (MAI), with a perovskite film created via thermal annealing. Using this technique PSCs having an active-area 1 cm^2^ were created having a PCE of 13%. Mesoscopic PSCs based on spray-deposited TiO_2_ scaffolds have also been demonstrated^26^. However, comparably few examples of spray-coated inverted architecture PSCs exist^16, 27^.

To develop a practical manufacture process to fabricate large-area perovskite PV, it is imperative that all layers within the device should be deposited via a scalable technique (ideally on a flexible substrate making it compatible roll to roll processing). However, this has only been demonstrated in a few cases, with most studies using inflexible glass substrates. One study of note fabricated devices in which all layers were deposited by doctor-blading (excluding the vacuum-processed back contacts) having an average PCE of over 10%^28^. This value was however reduced to 3.4% when the electrode was instead printed^14^. Recently, we reported on spray-coated planar inverted architecture PSCs where all solution-processed layers (namely PEDOT:PSS, perovskite and PCBM) were deposited by ultrasonic spray-coating, with an champion (average) PCE of 9.9% (7.1%) achieved^27^.

In this paper we extend our previous techniques, and use spray-coating to prepare all the layers in a mesoporous standard-architecture PSCs (except the contact electrodes), and create devices having improved performance and repeatability. Specifically, we spray-cast compact titania (cTiO_2_)^29–31^, mesoporous titania (mTiO_2_)^32^, a CH_3_NH_3_PbI_(3−x)_Cl_x_ precursor and doped spiro-OMeTAD layers, creating a champion cell having a PCE of 10%. We then utilise a range of microscopy and mapping techniques to explore the homogeneity and uniformity of the layers, and conclude that device efficiency is partially limited by (i) the presence of ~10 μm diameter aggregate defects in the spray-cast perovskite layer that act as local current shunts, and (ii) non-uniformities in the spiro-OMeTAD film that results in reduced charge carrier extraction and thus reduced fill-factor. We also explore our techniques to fabricate devices having an active-area of 1.5 cm^2^, reaching an efficiency of 6.6%. As far as we are aware, this is the first example of ultrasonic spray-coating being used to deposit a doped spiro-OMeTAD hole-transport layer, as well as the first example of a multilayer spray-cast mesoporous PSC.

## Results

### Ultrasonic Spray-Coating

Spray-coating was carried out using a Prism ultrasonic spray-coating system supplied by Ultrasonic Systems, Inc. This instrument employs resonant oscillation of a piezo-electric tip to shear a coating ink into a fine mist of micron-sized droplets that are then directed to a surface of interest via a focused nitrogen gas jet^22^. Such “nozzle-less” techniques offer independent control of droplet formation and spray pattern. During the coating procedure, the spray-head is passed over the surface at a fixed height. From extensive optimization trials, we were able to adjust film thickness and drying rates *via* control of lateral head-speed, solution concentration and substrate surface temperature. Here, the formation of uniform thin-films is dependent on the ability of the ink to wet the surface. Unlike spin-coating, there are no lateral forces in a conventional spray-coater that act to spread a wet-film across a surface and ensure that droplets coalesce to form a continuous film. Instead, only capillary forces (which are relatively weaker) act to move liquid droplets across the substrate surface. This can lead to poor surface coverage, particularly if the solvent has a high surface tension or a low surface energy. The rate at which the film dries is also important; if the drying time is too short then the droplets can dry before forming a uniform wet film. Conversely if film drying occurs too slowly, then the wet film can undergo shrinkage^16, 33^ forming “coffee-ring” patterns^34^.

To address this, we have performed a detailed optimisation study in which we have developed a series of different ultra-sonic spray-coating processes and inks to deposit mesoporous TiO_2_, a MAI:PbCl_2_ precursor and a doped spiro-OMeTAD hole-extracting layer (see further details of ink formulations and deposition parameters in Table 1 in *Experimental Methods* section). For comparative purposes, the deposition of all layers was also explored by spin-casting. Small-area devices were fabricated on pre-patterned 15 × 20 mm glass-FTO substrates. The fabrication process commences with the deposition of a hole-blocking compact TiO_2_ layer (cTiO2) by spray pyrolysis using a hand-held spray-gun. Here, TAA (titanium diisopropoxide bis(acetylacetonate)) was diluted in isopropanol and sprayed onto FTO-glass placed on a hot-plate and held at 450 °C^35^ and then sintered for 1 hour. A mesoporous TiO_2_ (mTiO_2_) layer was then deposited at room temperature to act both as a scaffold for the perovskite layer and as an electron-accepting contact. Here, a mTiO_2_ paste was used that was diluted to 10 wt% and 22 wt% with ethanol for spray- and spin-casting respectively. After the evaporation of the ethanol, the samples underwent further sintering for 1 hour at 450 °C to harden the films into a dense mTiO_2_ scaffold ready for perovskite precursor deposition.

**Table 1:** Summary of thin-film deposition protocols (*) refers to substrate temperature during ink deposition.

Parameter	mTiO_2_		Perovskite		Doped Spiro-OMeTAD	
	spin	spray	spin	spray	spin	spray
substrate temperature*	Ambient	Ambient	Ambient	55 °C	Ambient	40 °C
*annealing*	1 hr min @ 450 °C	1 hr min @ 450 °C	45 minutes @100 °C	45 minutes @ 100 °C	none	None
*speed*	3000 rpm/30 s	60 mm s^−1^	2000 rpm/30 s	200 mm s^−1^	2000 rpm/30 s	150 mm s^−1^
*ink conc*	22 wt%	10 wt%	630 mg ml^−1^	450 mg ml^−1^	96 mg ml^−1^	45 mg ml^−1^
*solvent*	Ethanol	Ethanol	DMF	DMF	CB	1:1 CF:CB
*ink temp.*	Ambient	Ambient	Ambient	Ambient	Ambient	Ambient

The precursor perovskite films (2.95:1.00 MAI:PbCl_2_ solution containing 1% (by volume) hydrogen iodide (HI) in DMF were then coated on the FTO/cTiO_2_/mTiO_2_ surface under ambient lab conditions maintained at (20 ± 2)°C and (30 ± 5)% RH. Here, the role of the HI additive was to improve the solubility of PbCl_2_ and increase the surface coverage of the final perovskite film^36^. This precursor was spray-cast using our previously-described methods used to fabricate inverted-architecture PSC devices^16, 27^. Here the substrate is heated to 55 °C during deposition to replicate the drying dynamics that occur during spin-coating^37^. After deposition, the samples were transferred to a secondary hotplate held at 100 °C for 45 minutes to convert the precursor film to a MAPbI_3−x_Cl_x_ perovskite.

Spiro-OMeTAD films were prepared by both spin- and spray-casting. For spray-casting, we developed a mixed solvent system (1:1 chlorobenzene:chloroform) to deposit doped spiro-OMeTAD. This exploited solvent surface tension gradient induced flows (Marangoni effect) to produce favourable spreading capabilities^38, 39^, resulting in the formation of a relatively smooth film. It was found however that the addition of dopants (Li-TFSI, TBP, and FK209) to the spiro-OMeTAD solution (conventionally used to increase the conductivity of the final film) had the unwanted effect of increasing ink surface tension which suppressed its wetting and spreading properties (see *Supplementary Information*). To address this issue, we found that the addition of a small quantity (0.003 mg mL^−1^) of a high molecular weight (M_N_ ~ 8 MDa) polyethylene glycol polymer (PEG) to the ink increased its viscosity through chain entanglement effects^40^. Using this formulation, we were able to deposit films having improved uniformity (*see* Figure S1).

**Figure 1:**
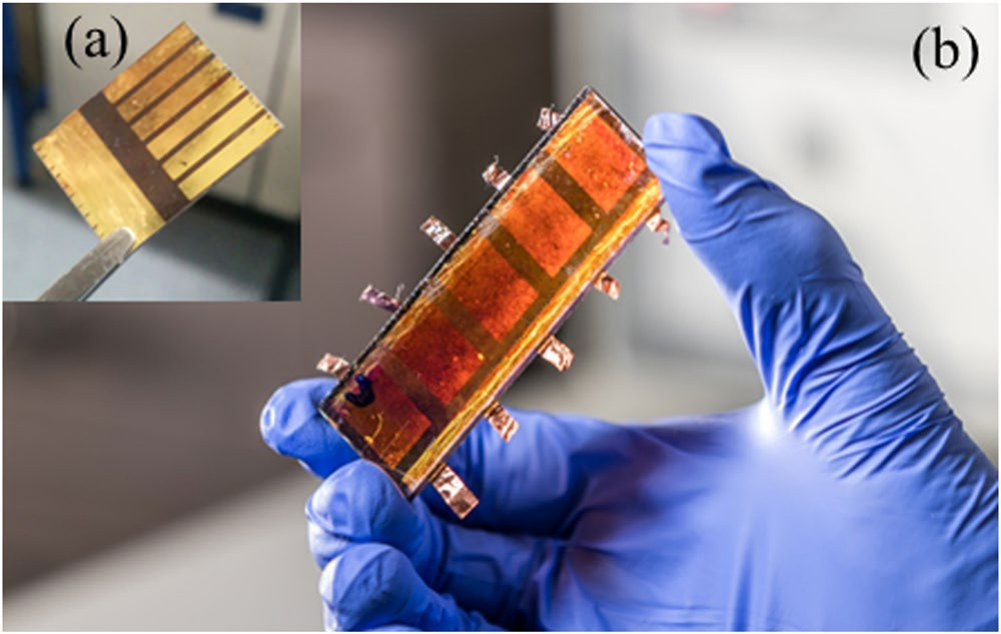
Part (**a**) and (**b**) show images of completed small-area and large-area PSC devices respectively.

Finally, to create a PSC device, patterned gold counter electrodes were vacuum evaporated onto the Spiro-OMeTAD surface, creating six independent cells. Each cell had an active-area of 4 mm^2^ whose size was defined by the overlap of anode and cathode stripes. In order to evaluate effects associated with scaling-up of our spray-deposition protocols, we used the same spray-casting methodology to create large-area PSC devices on 25 × 75mm FTO/glass slides. Here, five independent large-area PSCs were fabricated, with each device having an active-area of 1.51 cm^2^. Images of completed all-spray-cast PSCs having an active-area of 4 mm^2^ and 1.51 cm^2^ are shown Fig. 1.

Devices were characterized by measuring their J-V curves under 1 Sun AM1.5 G simulated solar illumination (see *Experimental Methods*). Note that although six PSC devices were fabricated on each small-area substrate, the two devices at the edge of the substrate were omitted from our analysis due to defects associated with film formation at this location. We have also used laser-beam induced imaging (LBIC) to explore the homogeneity of photocurrent generation. In this technique, 405 nm light from a diode-laser was focused to a point and raster scanned across the surface in 25 µm step-sizes, with the photocurrent recorded using a pico-ammeter. Scanning profilometry using a Bruker DektakXT having a vertical and lateral spatial resolution estimated to be 1 nm and 12.5 µm (defined by the tip radius) respectively were also used to obtain topographic images of the surfaces of individual layers at various stages in the device fabrication process.

### Device and Film Characterisation

**Figure 2:**
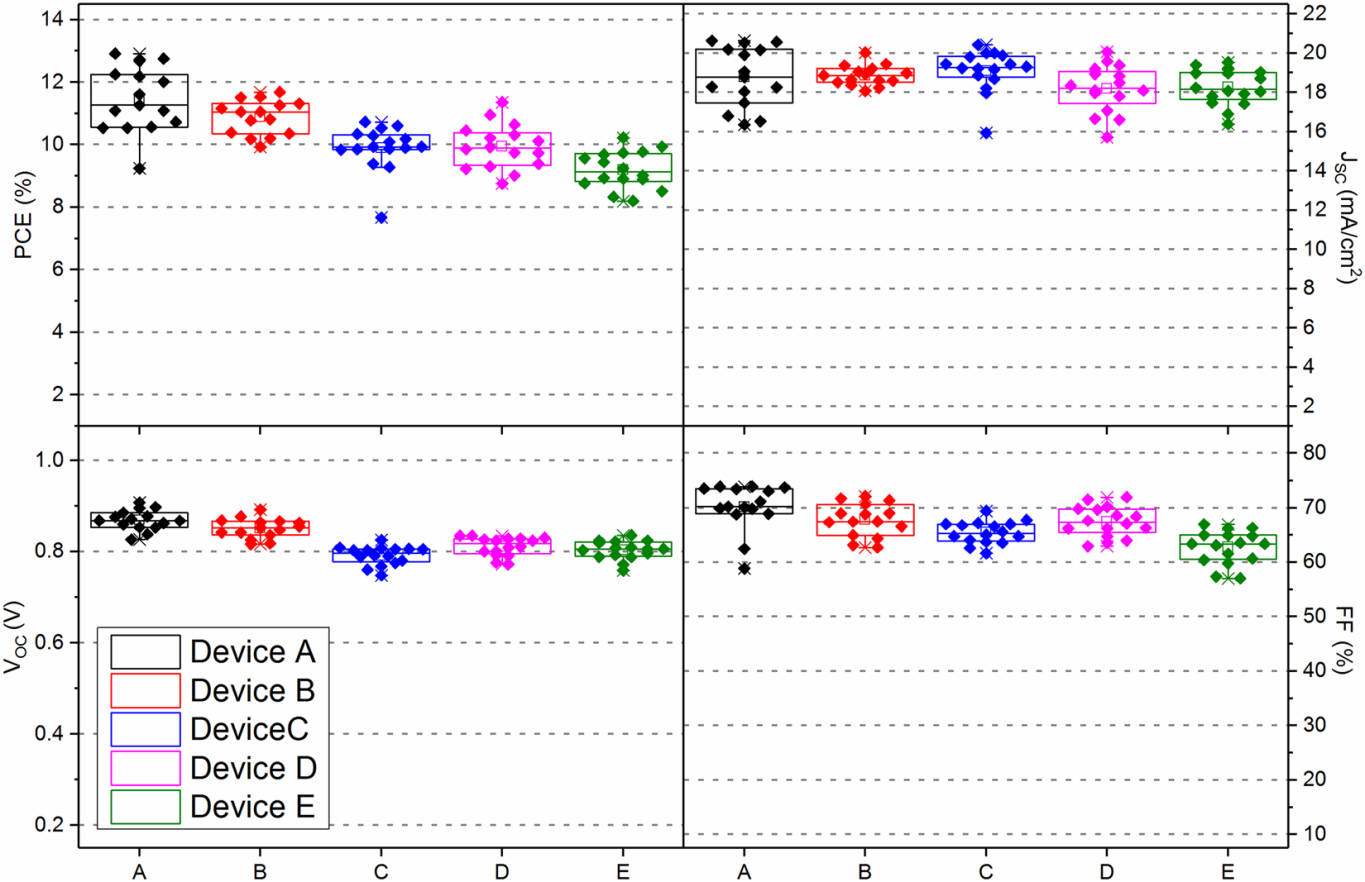
Box plots showing statistical data of PSC performance from devices A–E (see Table 2 for a description of device labels).

**Figure 3:**
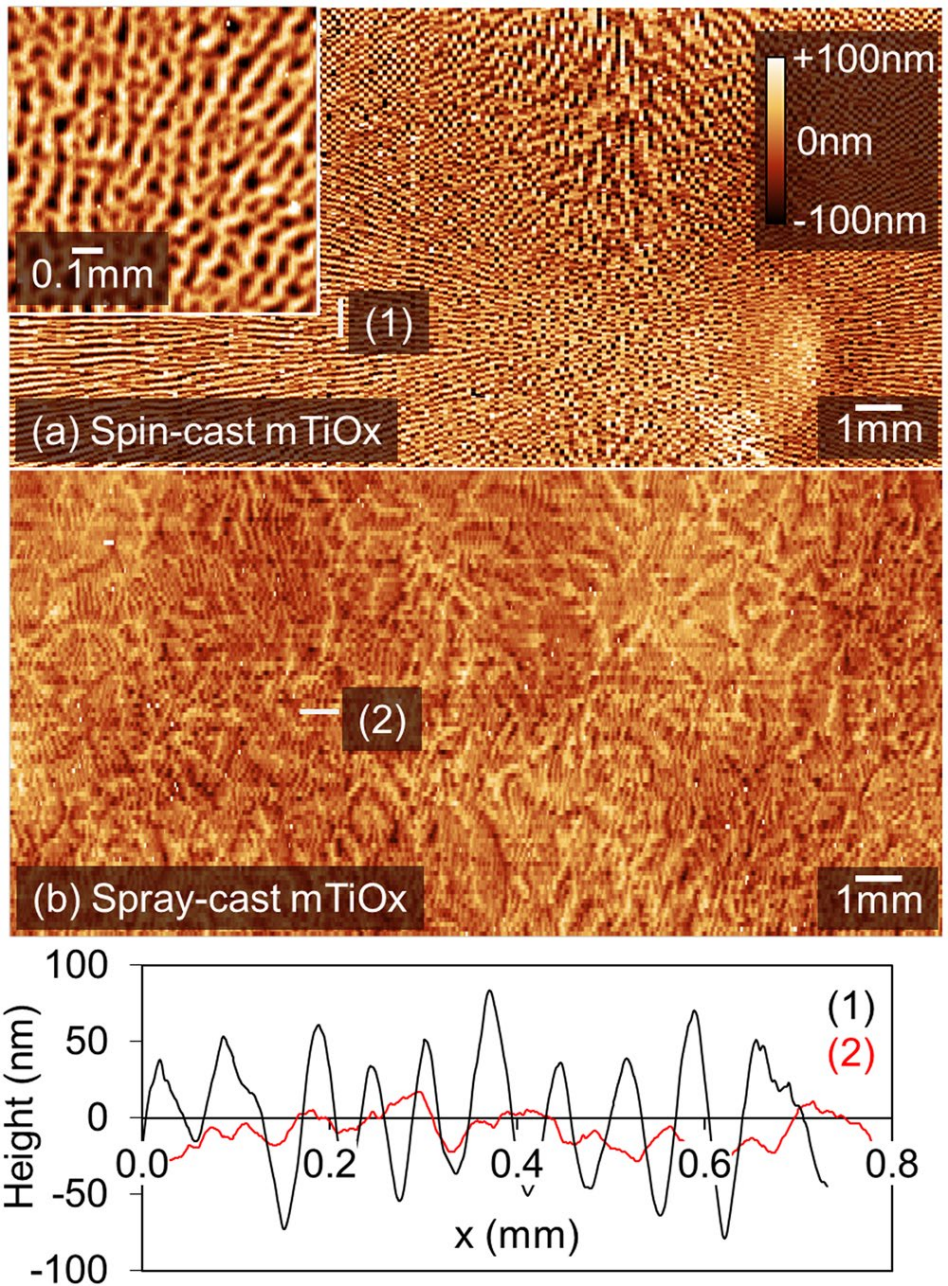
Topographic images of mTiOx prepared on a glass/FTO/cTiOx surface. Part (**a**) shows a spin-cast film with a high-resolution map shown in the inset. Part (**b**) shows surface topography of a spray-cast film, with line profiles determined from sections labelled (1) and (2) plotted using black (spin-cast) and red (spray-cast) lines shown as an inset. All images are plotted on the same colour scale.

We first present a summary of performance metrics from our small-area device fabrication study in Table 2, together with statistical data recorded from 16 independent cells in a box plot in Fig. 2. Here, device A was fabricated by spin-coating all layers and had a PCE of (11.4 ± 1.0)%. On spray-casting the mTiO_2_ scaffold (device B), we find that the PCE is slightly reduced at (10.9 ± 0.5)%, however the statistical significance of this reduction is low. Here, any reduction in average PCE results from a drop in V_OC_ from (0.87 ± 0.02) V to (0.85 ± 0.02) V, and in FF from (70 ± 4)% to (68 ± 3)%. Such differences appear to result from changes in the mTiO_2_ thickness that appears dependent on the nature of the technique used in its deposition. This is illustrated in Fig. 3(a) and (b) respectively, where we plot topographic images of spin and spray-cast mTiO_2_ thin-films after sintering. Interestingly, we find that the spin-cast film shows strong evidence of solutal Marangoni effects^41, 42^ whereby surface tension gradients cause a flow of the material to highly concentrated areas. Such effects area characterised by striations^43^ in film thickness that appear as periodic features (70 ± 1) µm in the cross-sectional thickness data (*see* Fig. 3(b) inset). This results in much larger peak-to-valley thickness variations in the spin-cast film surface compared with its spray-cast analogue (90 nm compared with 20 nm respectively). Because of this, one might anticipate superior performance from device B owing to its improved mTiO_2_ uniformity, however this is not observed. We are unable to account for the reduction in fill factor responsible for this; however we note that the efficiency of mesoporous standard architecture PSCs is very sensitive to differences in mTiO_2_ film thickness, and the efficiency variations may simply reflect uncertainties associated with film thickness and measurement.

**Table 2:** A summary of PSC performance metrics and deposition technique used to fabricate each layer.

	Device A	Device B	Device C	Device D	Device E	Device E
Area (cm^2^)	0.026	0.026	0.026	0.026	0.026	1.008
*mTiO*_2_	Spin	Spray	Spin	Spray	Spray	Spray
*Perovskite*	Spin	Spin	Spray	Spray	Spray	Spray
*HTM*	Spin	Spin	Spin	Spin	Spray	Spray
PCE (%)	**12.9** (11.4 ± 1.0)	**11.7** (10.9 ± 0.5)	**10.7** (9.9 ± 0.7)	**11.4** (9.9 ± 0.7)	**10.2** (9.2 ± 0.6)	**6.9**
J_SC_(mA/cm^2^)	**20.6** (18.8 ± 1.5)	**20.0** (18.9 ± 0.5)	**20.4** (19.1 ± 1.0)	**20.1** (18.2 ± 1.2)	**19.5** (18.2 ± 0.9)	**18.6**
V_oc_ (V)	**0.91** (0.87 ± 0.02)	**0.89** (0.85 ± 0.02)	**0.82** (0.79 ± 0.02)	**0.83** (0.81 ± 0.02)	**0.84** (0.80 ± 0.02)	**0.83**
FF (%)	**74** (70 ± 4)	**72** (68 ± 3)	**69** (65 ± 2)	**72** (67 ± 3)	**67** (63 ± 3)	**45**

Bold is the peak value with the average and standard deviation presented in parenthesis.

We now turn our attention to the perovskite absorber layer. Here, we find that there is a reduction in efficiency associated with spray-casting this layer in particular. Returning to Table 2, we compare devices A and C in which the perovskite precursor layer was either spin- or spray cast (with all other layers being spin-cast). Here, we find a significant reduction in PCE from (11.4 ± 1.0)% to (9.9 ± 0.7)% as a result of spray-casting the perovskite layer. This effect is also evident when comparing devices B and D. Here, both devices employ spray-cast mTiO_2_ and spin-cast spiro-OMeTAD layers, with the perovskite precursor layer being spin-cast in device B and spray-cast in device D. Here, we find a reduction in efficiency as a result of spray-casting, with efficiency dropping from (10.9 ± 0.5)% to (9.9 ± 0.7)%; an effect that is almost entirely accounted for by losses in V_OC_.

**Figure 4:**
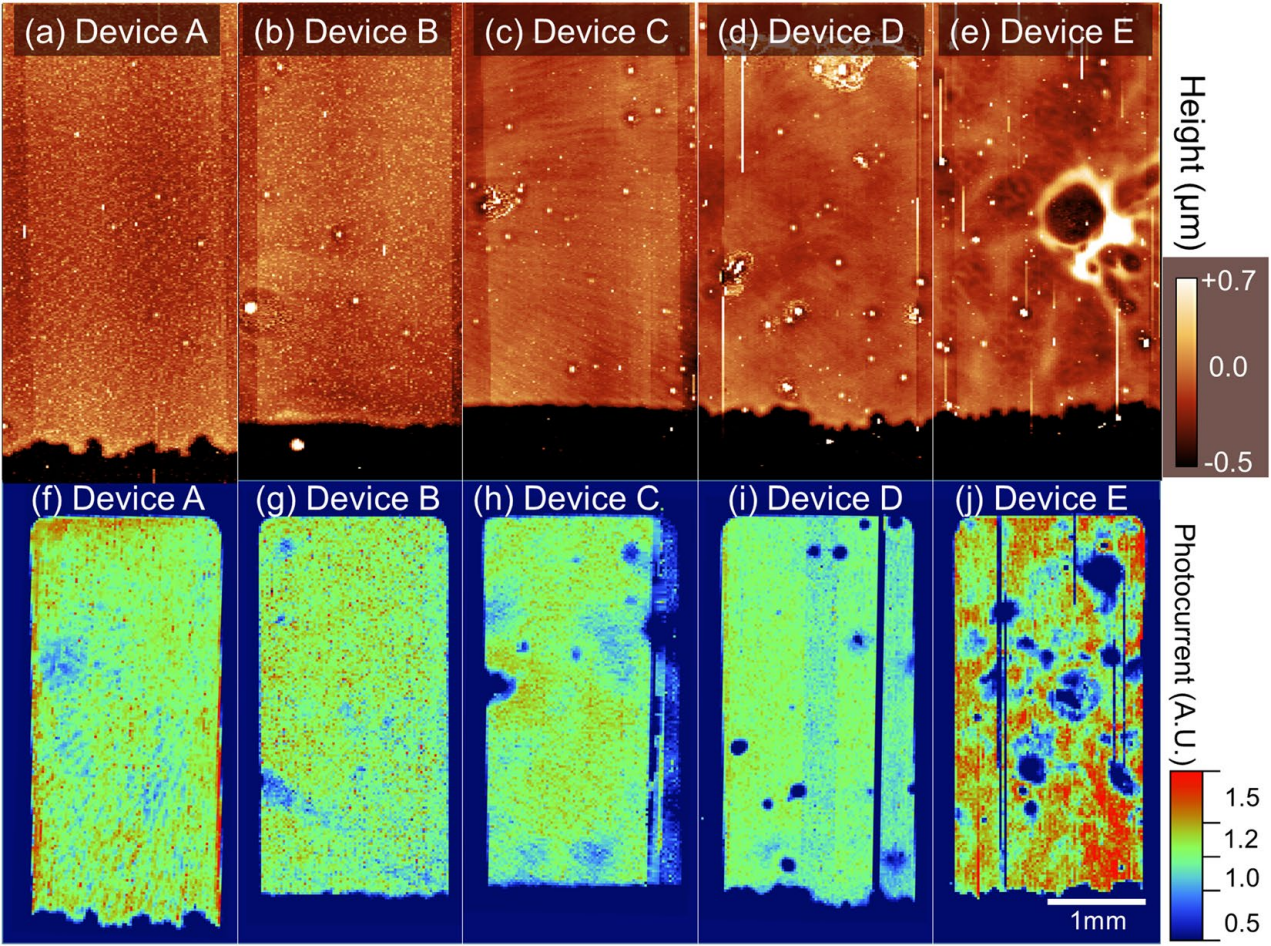
Topographic images of device A–E (parts (**a–e**)) measured by scanning profilometry with colour and lateral scales shown inset in part (**a**) and (**e)** respectively. LBIC (laser beam induced current mapping) maps of Devices A to E shown in parts (**f**) to (**j**). Data has been normalised to the average photocurrent and represented on the same colour scale. All images are plotted on the same lateral scale shown inset in part (**j**).

Once again, we use surface profilometry to understand such effects. In Fig. 4(a) to (e), we plot topographic images of devices A to E (image recorded over the surface of the gold anode and surrounding region). Here the active-area of each device can be recognised *via* the raised rectangular region that corresponds to the evaporated gold film, with dark region in the lower part of each image corresponding to the edge of the etched FTO. Faint striations consistent with the underlying spin-cast mTiO_2_ topology are evident in device A [Fig. 4(a)] and device C [Fig. 4 (c)] as expected. It is also apparent that small raised features (defects) having a lateral diameter of 10 to 40 µm and height of up to 25 µm are observable in all images.

To characterise such defects, we threshold image data recorded from device A to D at 1 µm and perform a particle size analysis characterising both particle height and number density^44^ (*see* Figure S2). From these plots and the images shown in Fig. 4, it is apparent devices in which the perovskite precursor film is spray-cast are characterised by a greater density of defects (see Table 3). Note, that most defect particles imaged using the surface profiler had an apparent in-plane diameter of around 10–15 μm. This value however is coincident with the spatial resolution of the surface profiler, indicating that the diameter of many of the defects is likely to be smaller than this. To determine the typical size of such defects, we have used an optical microscope to image the surface of a spray-cast perovskite film, with a typical image shown in Figure S7. An analysis of such images suggests that the defect structures indeed have a diameter of around 10 μm.

**Table 3:** Results of particle size analysis carried out on data from surface topographs shown in Fig. 4 (a) to (d).

	Device A	Device B	Device C	Device D
Defects per 1 cm^2^	100	140	320	420
Height (µm)	2 (5)	4 (24)	2 (12)	3 (14)

Average values are shown outside and maximum values inside parentheses. Data was thresholded at 1 µm and image area remained fixed at 13.5 mm^2^.

**Figure 5:**
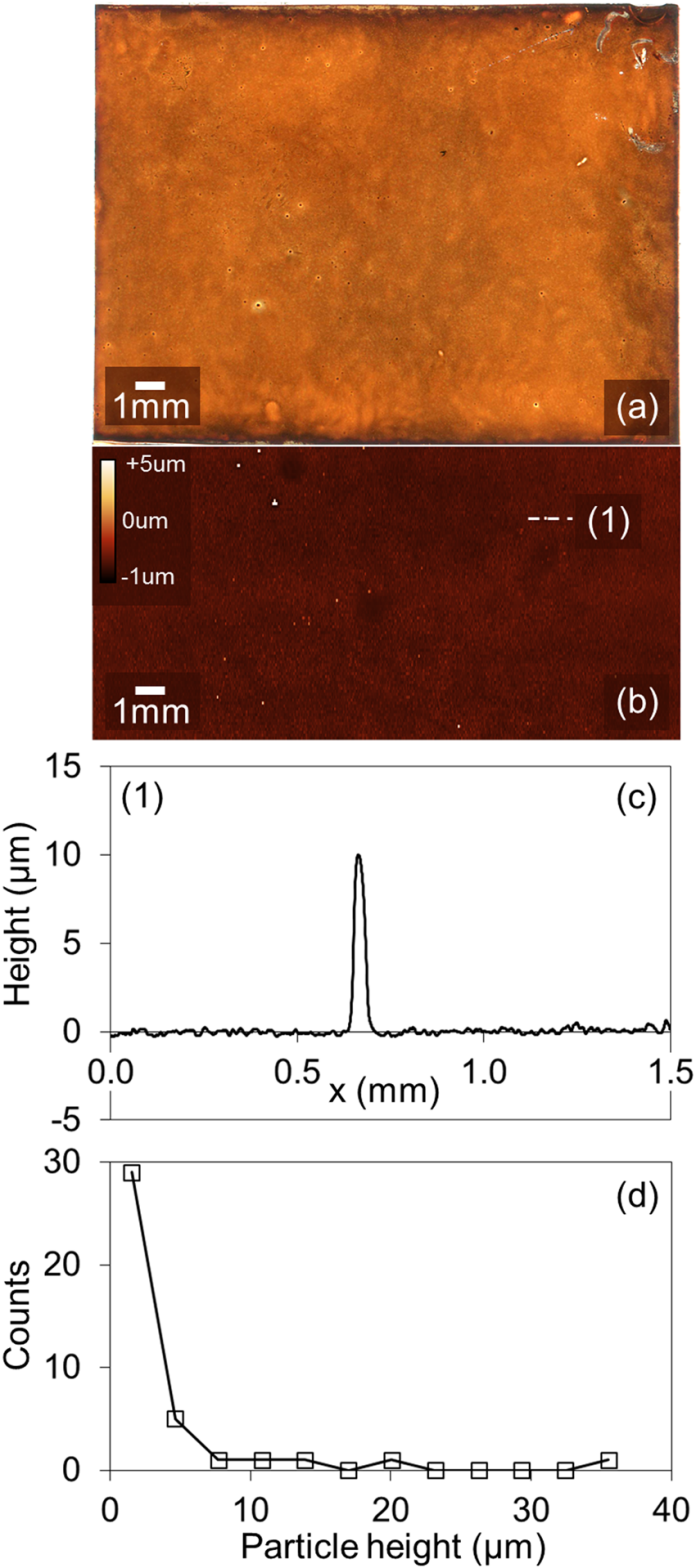
Part (**a**) shows an optical transmission image of a FTO/cTiOx/mTiOx/perovskite film prepared by spray-coating. Part (**b**) shows a representative topographic image of the film shown in part (**a**), however these images do not correspond to the same location on the film surface. Part (**c**) shows a cross-section recorded through one of the aggregates (visible as white-spots) in part (**b**). The location at which this data was recorded is shown using a dotted line. Part (**d**) plots a histogram of particle height determined from an analysis of the film recorded over an area of 5 × 10 mm^2^.

To confirm that film defects seen in Fig. 4 are associated with the perovskite film, we have studied FTO/cTiO_2_/mTiO_2_/perovskite surfaces prepared by spray-coating. In Fig. 5(a) we show an optical image of the film recorded in transmission, with part (b) showing a topography map of a representative area of the film with both images plotted on the same scale. It can be seen that the optical image [Fig. 5(a)] is characterised by a series of dark spots that are apparently consistent with aggregate-like defects that are visible as white-spots in the representative topography map [*see* Fig. 5(b)]. We plot a cross-section recorded through a single aggregate-defect in the Fig. 5(c) where it can be seen that the height of such defects is indeed much greater than the thickness (and normal roughness) of the perovskite film. Notably, such defects are not observed in spin- or spray-cast cTiO_2_ or mTiO_2_ layers. An analysis of the height distribution of these aggregates [(*see* Fig. 5(d)] indicates a distribution of particle sizes having an expectation value of 1.8 µm and variance of 3.2 µm.

At this point, we are unable to assign the origin of these defects, however we speculate that they are in fact PbCl_2_ aggregates. We base this conclusion on the fact there is a large difference in relative solubility of PbCl_2_ and MAI, and that PbCl_2_ may undergo local aggregation or crystallisation during film drying as a result of fluctuations in local material concentration. It is unclear why larger aggregates apparently appear in the spray-cast films when their drying time is in fact shorter than the spin-cast analogues (15 *vs* 30 s respectively). We suspect that films that are spin-cast are subject to shear forces that constrain the film surface^45^ and reduce any tendency for the creation of compositional concentration gradients that lead to the formation of aggregates. In spray-casting however, such shear forces are absent, with convective flows due to the heated substrate possibly driving lateral material flow across the surface^37^ even though the overall drying time is shorter in the latter. We speculate therefore that such effects are responsible for the increased density of aggregates found in spray-cast films. Such aggregates in the perovskite film most likely result in charge-carrier leakage pathways through the top spiro-OMeTAD hole-transport layer that is (400 ± 10) nm thick in devices A–D. This leads to additional charge-carrier recombination losses that act to reduce V_OC_ and reduce device performance compared to their spin-cast analogues.

Finally, we discuss the spray-deposition of spiro-OMeTAD. Here, we again saw a reduction in device performance on spray-casting this layer as shown in Table 2. We now compare device D and E in which TiO_2_ and perovskite-precursor films were deposited by spray-casting, but spiro-OMeTAD films were spin and spray-cast respectively. It can be seen that on spray-casting, the device PCE reduces from (9.9 ± 0.7)% to (9.2 ± 0.6)% as a result of a reduction in FF from (67 ± 3)% to (63 ± 3)%. We ascribe this reduction to a general decrease in HTM layer uniformity; a process that leads to a concomitant increase in series resistance. Despite the reduction in efficiency resulting from spray-coating the perovskite-precursor and spiro-OMeTAD layers, it can be seen that device E in which all layers were spray-cast has an average PCE of (9.2 ± 0.6)%. This represents a marked enhancement in performance (with a narrowed spread in device performance) compared to our previous study on all-spray inverted PSCs^27^ in which we obtained an average PCE of (7.1 ± 1.7)%. For completeness, we include EQE spectra recorded from champion all spin (A) and all spray (E) devices in Supplementary information Figure S6. The reduction in the homogeneity of devices incorporating a spray-cast spiro-OMeTAD film can be seen in Fig. 4(e) (corresponding to device E) where large variations in height are evident. This is likely responsible for the reduced fill factor for these devices. Indeed, we found that the spray-deposition of doped spiro-OMeTAD to be very challenging, however highly uniform films of *undoped* spiro-OMeTAD could be prepared by spray-coating without apparent difficulty. We suspect that the presence of ionic dopants such as LiTFSI and FK209 may increase the surface tension of the spiro-OMeTAD ink and therefore adversely impact its wetting properties as a result of increased surface tension (*see* Figure S1(c)). This is likely to impede droplet coalescence and thus causes solution dewetting. However the uniformity of the doped spiro-OMeTAD film can be significantly improved through the addition of a PEG rheology modifier, which we found enhanced the performance of resultant PSC devices (*see* Figure S3).

### Laser Beam Induced Current Mapping

To further characterise the uniformity of our PSCs and explore spray-cast film properties we have used Laser Beam Induced Current (LBIC) mapping. This powerful diagnostic technique can be used to create a spatial map of photocurrent homogeneity. This is shown in Fig. 4 where we plot LBIC maps recorded from devices A to E (shown in parts (f) to (j) respectively). It is immediately apparent that the efficiency of photocurrent generation across devices A and B (corresponding to devices in which the perovskite-precursor is spin-cast), is highly uniform and only varies by around 8% over length-scales of a few mm. Conversely, PSCs containing spray-cast perovskite precursors (devices C and D), are characterised by less uniform photocurrent generation, varying by 16 and 21% respectively. Interestingly in device D, we observe isolated regions having a diameter of (110 ± 10) µm that are characterized by a low photocurrent. By comparing topographic and LBIC images (Fig. 4 (a) to (j) respectively) we find that regions of low photocurrent closely correlate with the large aggregate-type defects associated with perovskite spray-deposition.

It is also apparent that there are periodic (radial) features visible in the LBIC images recorded from devices A and C that were also apparent in the topography images shown in Fig. 4. We conclude therefore that the thickness variations in the mTiO_2_ play a significant role in determining the efficiency of photocurrent generation, and that accurate control over this layer is of key importance for effective device optimisation. Notably such features are not observable in the LBIC images recorded from devices B and D due to the improved uniformity of the spray-cast mTiO_2_ layer. The effect of the non-uniform spray-cast spiro-OMeTAD film on photocurrent generation in device E is clearly apparent in Fig. 4(j), and in the photocurrent histogram and cross-sectional data (*see* Figs S4 and 5). Here, the photocurrent varies by as much as 22% across the surface of the device, indicating the importance of developing improved processing protocols for this layer. For completeness, we also present cross-sectional SEM images recorded from devices A and E in Supplementary Information, Figure S8. This confirms the results presented in Fig. 4, with enhanced non-uniformity across both the perovskite and spray-cast spiro-OMeTAD being evident.

### Large-Area Device

**Figure 6:**
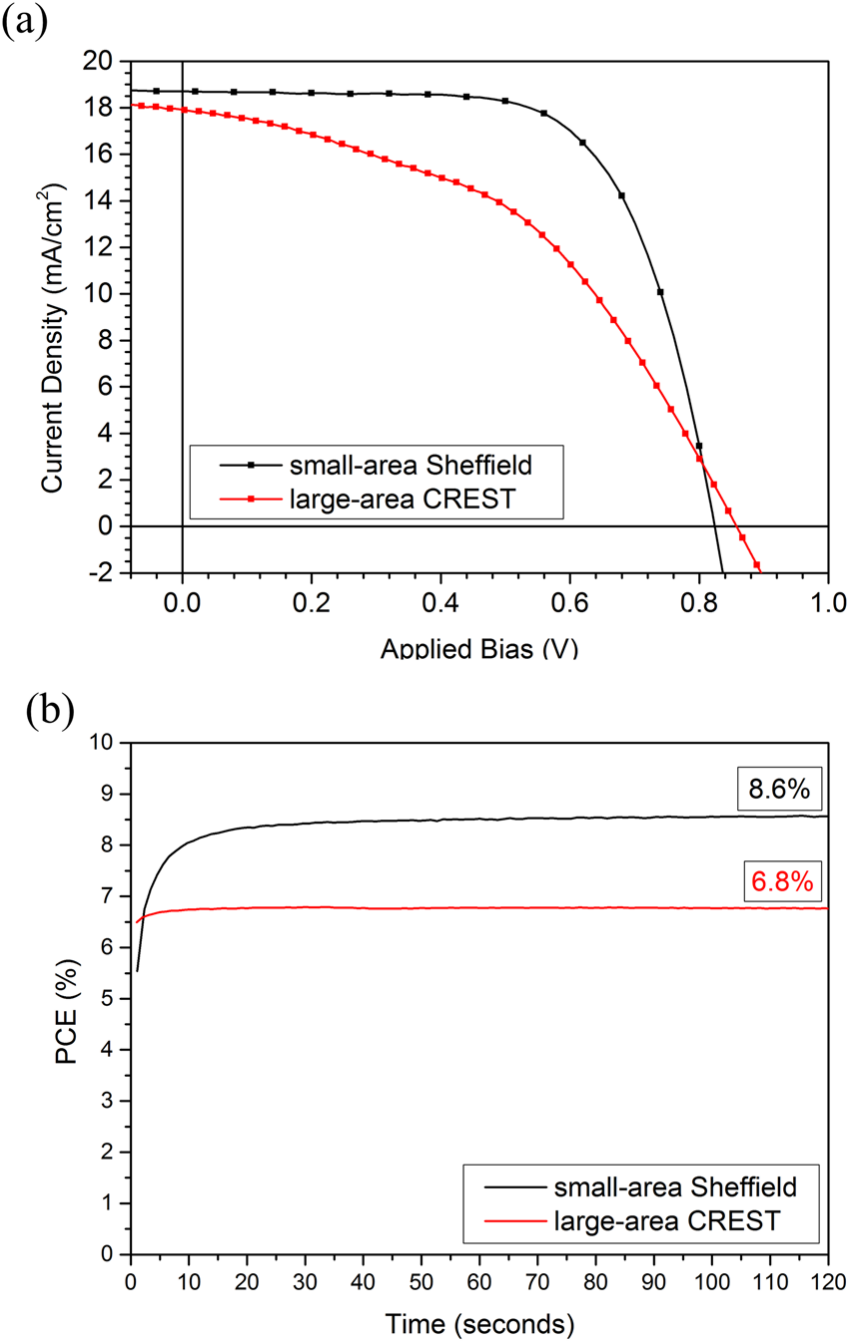
Part (**a**) shows the champion reverse scan JV characteristics for the small-area (measured in Sheffield) and large-area devices (measured at CREST). Part (**b**) shows the stabilised PCE for the champion devices held at a fixed voltage around the maximum power point.

In order to evaluate the scalability of our spray-casting deposition protocols, we have fabricated large-area cells on 25 × 75 mm FTO/glass slides. Here, all solution processable layers were deposited via spray-coating using the techniques developed to fabricate device E. Again, the devices utilised a thermally evaporated gold contact to define five independent cells, each having an active-area of 1.51 cm^2^ (as shown Fig. 1(b)). To gain additional confidence in our device test protocols, we have also recorded *JV* characteristics of devices having an active-area of 1.51 cm^2^ using a solar simulator at CREST, UK. These measurements (performed through a 1 cm^2^ aperture mask) confirmed a device PCE of (6.59 ± 0.16)%; a value in good accord with measurements recorded using the solar simulator in Sheffield. A *JV* scan from the champion large/small-area devices with a corresponding stabilised PCE measurement is plotted in Fig. 6 (for more JV data see S6). It is clear that J_SC_ and V_OC_ are largely unaffected by scale-up which demonstrates the robustness of our process. However there is a significant reduction in FF from 67% to 45% that leads to a loss in PCE associated with scale-up from 10.2% to 6.9%. This reduction in PCE results from parasitic losses as a result of increased series resistance associated with longer FTO channel lengths which tend to increase with the device area^46^.

## Discussion

We have developed a method to fabricate multilayer standard architecture perovskite solar cells in which all solution processible layers (cTiO_2,_ mTiO_2_, perovskite absorber and doped spiro-OMeTAD) were deposited by spray-casting. We show that this method can be used to fabricate cells with a peak PCE of over 10% and an average of 9.2%, with a relatively low distribution in cell performance (σ = 0.6). This result compares favourably with devices in which all layers were deposited by spin-casting, where devices had an average PCE of 11.4%. Note that the baseline efficiencies of spin-cast devices demonstrated here are around a factor of two lower than state of the art devices that are processed using different perovskite formulations in an inert water and oxygen-free environment. The reduced efficiencies reported here result from the fact that all processing steps here were performed in air; a condition that is likely to be beneficial when developing a low-cost industrial process. However we expect that higher efficiency devices will be possible by transferring our process to a spray-coater housed within a nitrogen filled glove-box. Using laser beam induced photocurrent mapping and optical microscopy, we attribute the reduction in performance associated with spray-casting (compared to spin-casting) to the presence of micron-sized defects in spray-cast perovskite films that reduce V_OC_ through charge-carrier recombination losses, and significant film-thickness fluctuations in the spray-cast spiro-OMeTAD films that reduce FF by series resistance losses. We also explore the suitability of this process to fabricate larger-area devices, and fabricate fully spray-cast cells having an active-area of 1.5 cm^2^. These were characterised using a solar simulator at CREST, where a device PCE of (6.59 ± 0.16)% was determined. This reduction in PCE on scale-up resulted from parasitic losses caused by increased serial resistance of the FTO electrode.

## Methods

### Device fabrication

Small- and large-area devices were fabricated on TEC 10 and TEC 8 FTO/glass substrates (XOP glass) respectively. Substrates were etched with zinc powder and 4 M HCl before being sonicated with Helmanex detergent solution, deionised water, and IPA. All device steps reported below were conducted under ambient lab conditions (in air) unless otherwise stated. All solvents used in this research were purchased from Sigma.

Substrates were firstly transferred to a hotplate where spray-pyrolysis was performed. 1.72 mL of titanium diisopropoxide bis(acetylacetonate) (Sigma 325252) was diluted with IPA to 20 mL. This was then sprayed onto the substrates held at 450 °C *via* a handheld spray gun (Draper 09709) with a nitrogen feed at 30 psi. Substrates were coated every 30 seconds until all the precursor was used. These were then left to sinter for 30 minutes.

Spray coating was performed using an Ultrasonic Systems Inc. Prism 300 system. During coating, the ultra-sonic tip was positioned 60 mm above the substrate surface and vibrated at 35 kHz while fluid from a coating reservoir was fed to the tip. This dispersed the ink into micron-sized droplets that were directed to the surface using a carrier gas whose pressure was set to 10 psi giving a wide spray pattern (*ca* 50 mm). During spraying, the spray head was scanned a lateral distance of 150 mm over the device substrates in a single pass. Note that the width of the spray-pattern was wider than the individual device substrates (25 mm), and thus significant heterogeneity across the spray-mist pattern at the sample surface is not anticipated. Between coating processes, pure solvent was flushed through the ink delivery system before the next ink reservoir was refilled. Substrates were mounted on a hotplate at elevated temperature in order to control the film drying-rate.

Mesoporous titanium oxide paste (18-NRT Dyesol) was diluted to 22 wt% in ethanol for spin-coating, and 10 wt% for spray-coating. The paste was spin coated at 3000 rpm. The spray parameters were as follows: fluid pressure 60 mbar, head velocity 60 mm s^−1^ and substrate temperature 22 °C. After deposition, the substrates were sintered for 1 hour at 450 °C.

Perovskite precursor ink was prepared using a stoichiometric ratio of 2.95:1.00 MAI (Ossila) to lead chloride (99.999%). Precursor inks were prepared at 630 mg ml^−1^ in DMF containing 1 v% hydroiodic acid. This precursor was the spin-coated at 2000 rpm to create thin films. For spray-coating the precursor ink was diluted with DMF to 450 mg/ml and deposited using the following parameters: fluid pressure 50 mbar, head velocity 200 mm s^−1^, and substrate temperature 55 °C. After deposition, substrates were annealed at 100 °C for 45 minutes to convert them to a perovskite.

A stock Spiro-OMeTAD solution (Ossila) was prepared at a concentration of 96 mg mL^−1^ in chlorobenzene. This material was then doped by adding the following quantities of dopant to 1 mL of solution: 30 µl Li-TFSI (175 mg mL^−1^ in acetonitrile), 10 µl TBP, and 20 µl of FK-209 (175 mg mL^−1^ in acetonitrile). Films were then spin-cast onto the perovskite at 2000 rpm. For spray-coating, the doped solution was diluted to 45 mg mL^−1^ in chlorobenzene and chloroform such that the solvent ratio was 1:1. A small quantity of the polymer PEG (5 mg mL^−1^ in chlorobenzene) was added such that the PEG concentration was 0.003 mg mL^−1^. The spray parameters used to deposit the doped Spiro-OMeTAD solution were as follows: fluid pressure 20 mbar, head velocity 150 mm/s and substrate temperature of 40 °C.

Finally an 80 nm gold top contact was evaporated in an Edwards Auto 306 bell-jar evaporator at a pressure of *ca* 10^−6^ mbar.

### Device characterisation

Devices were characterised by measuring their J-V curves under AM1.5 simulated solar irradiance. When testing a large- and small-area cells the illuminated area was defined through a shadow mask having an aperture of 1.0077 and 0.026 cm^2^ respectively. Devices were tested under ambient conditions using a Newport 92251A-1000 solar simulator. An NREL certified silicon reference cell was used to calibrate the simulated AM1.5 G light-output to 100 mW cm^−2^. A Keithley 237 source measure unit was then used to perform J-V measurements. During testing devices were swept from −1.2 V to +1.2 V, and then back to −1.2 V at a scan speed of 0.4 Vs^−1^. Performance metrics were extracted from the reverse J-V scan. Stabilised power measurements were performed on the cells by holding them at a fixed voltage and recording the current over the course of a few minutes.

The champion large-area solar-cell was taken to CREST for testing using a WACOM solar simulator. Details of this test are included in the supplementary information. EQE measurements were performed using a custom-built setup. Devices were illuminated with light from a 100 W tungsten-halogen light source coupled to a monochromator (Spectral Products DK240 1/4 m). The photocurrent was recorded with an Ossila Xtralien × 100 source measure unit. The photocurrent from the device under test was compared to a reference silicon photodiode (Newport) with a known spectral response to calculate the EQE.

### Dektak and LBIC measurements

Laser beam induced current (LBIC) maps were performed using a custom-built setup. A 3 mW 405 nm diode laser was passed through a spatial filter before being focused to a power density of 27 W cm^−2^. The sample was mounted on a computer-controlled XY-stage and moved in a sawtooth pattern. To map the sample, the beam was focused ***via*** a 10X infinity-corrected objective lens to a spot size of ***ca*** 10 μm and the stage was moved in 25 μm steps. The PSC photocurrent was collected using a Keithley 2400 source measure unit.

Surface topographs were measured with a Bruker Dektak:XT profilometer in map scan mode (12.5 µm tip radius, 3 mg stylus force) over an area of 2.7 × 5.0 mm^2^ with 25 (slow-scan) and 0.83 µm (fast-scan axis) step-size respectively.

## Electronic supplementary material

Supplementary Information

## References

[1] Kojima A, Teshima K, Shirai Y, Miyasaka T (2009). Organometal Halide Perovskites as Visible-Light Sensitizers for Photovoltaic Cells. Journal of the American Chemical Society.

[2] Yang WS (2015). High-performance photovoltaic perovskite layers fabricated through intramolecular exchange. Science.

[3] Green MA, Ho-Baillie A, Snaith HJ (2014). The emergence of perovskite solar cells. Nature Photonics.

[4] Xing GC (2013). Long-Range Balanced Electron- and Hole-Transport Lengths in Organic-Inorganic CH3NH3PbI3. Science.

[5] Stranks SD (2013). Electron-Hole Diffusion Lengths Exceeding 1 Micrometer in an Organometal Trihalide Perovskite Absorber. Science.

[6] Sutton RJ (2016). Bandgap-Tunable Cesium Lead Halide Perovskites with High Thermal Stability for Efficient Solar Cells. Advanced Energy Materials.

[7] Eperon GE (2014). Formamidinium lead trihalide: a broadly tunable perovskite for efficient planar heterojunction solar cells. Energy & Environmental Science.

[8] Snaith HJP (2013). The Emergence of a New Era for Low-Cost, High-Efficiency Solar Cells. Journal of Physical Chemistry Letters.

[9] Green MA (2012). Radiative efficiency of state-of-the-art photovoltaic cells. Progress in Photovoltaics.

[10] Gong J, Darling SB, You FQ (2015). Perovskite photovoltaics: life-cycle assessment of energy and environmental impacts. Energy & Environmental Science.

[11] Yang LY, Barrows AT, Lidzey DG, Wang T (2016). Recent progress and challenges of organometal halide perovskite solar cells. Reports on Progress in Physics.

[12] Guo DP, Yu JG, Fan K, Zou HY, He BW (2017). Nanosheet-based printable perovskite solar cells. Solar Energy Materials and Solar Cells.

[13] Li S-G (2015). Inkjet printing of CH3NH3PbI3 on a mesoscopic TiO2 film for highly efficient perovskite solar cells. Journal of Materials Chemistry A.

[14] Schmidt TM, Larsen-Olsen TT, Carle JE, Angmo D, Krebs FC (2015). Upscaling of Perovskite Solar Cells: Fully Ambient Roll Processing of Flexible Perovskite Solar Cells with Printed Back Electrodes. Advanced Energy Materials.

[15] Deng Y (2015). Scalable fabrication of efficient organolead trihalide perovskite solar cells with doctor-bladed active layers. Energy & Environmental Science.

[16] Barrows AT (2014). Efficient planar heterojunction mixed-halide perovskite solar cells deposited via spray-deposition. Energy & Environmental Science.

[17] Tait JG (2016). Rapid composition screening for perovskite photovoltaics via concurrently pumped ultrasonic spray coating. Journal of Materials Chemistry A.

[18] Huang HB (2016). Two-step ultrasonic spray deposition of CH3NH3PbI3 for efficient and large-area perovskite solar cell. Nano Energy.

[19] Das S (2015). High-Performance Flexible Perovskite Solar Cells by Using a Combination of Ultrasonic Spray-Coating and Low Thermal Budget Photonic Curing. Acs Photonics.

[20] Krebs FC (2009). Fabrication and processing of polymer solar cells: A review of printing and coating techniques. Solar Energy Materials and Solar Cells.

[21] Sondergaard RR, Hosel M, Krebs FC (2013). Roll-to-Roll fabrication of large area functional organic materials. Journal of Polymer Science Part B-Polymer Physics.

[22] Bose S, Keller SS, Alstrom TS, Boisen A, Almdal K (2013). Process Optimization of Ultrasonic Spray Coating of Polymer Films. Langmuir.

[23] Pham NP, Boellaard E, Burghartz JN, Sarro PM (2004). Photoresist coating methods for the integration of novel 3-D RF microstructures. Journal of Microelectromechanical Systems.

[24] Pham NP, Burghartz JN, Sarro PM (2005). Spray coating of photoresist for pattern transfer on high topography surfaces. Journal of Micromechanics and Microengineering.

[25] Liang Z (2015). A large grain size perovskite thin film with a dense structure for planar heterojunction solar cells via spray deposition under ambient conditions. Rsc Advances.

[26] Huang H (2015). Sprayed P25 scaffolds for high-efficiency mesoscopic perovskite solar cells. Chemical Communications.

[27] Mohamad DK, Griffin J, Bracher C, Barrows AT, Lidzey DG (2016). Spray-Cast Multilayer Organometal Perovskite Solar Cells Fabricated in Air. Advanced Energy Materials.

[28] Hwang K (2015). Toward Large Scale Roll-to-Roll Production of Fully Printed Perovskite Solar Cells. Advanced Materials.

[29] Chen, C., Cheng, Y., Dai, Q. L. & Song, H. W. Radio Frequency Magnetron Sputtering Deposition of TiO2 Thin Films and Their Perovskite Solar Cell Applications. *Scientific Reports* **5**, doi:10.1038/srep17684 (2015). 10.1038/srep17684 PMC4668551 26631493

[30] Zhang CX (2016). Influence of different TiO2 blocking films on the photovoltaic performance of perovskite solar cells. Applied Surface Science.

[31] Liang C (2017). Chemical bath deposited rutile TiO2 compact layer toward efficient planar heterojunction perovskite solar cells. Applied Surface Science.

[32] Sanzaro, S. *et al*. Multi-Scale-Porosity TiO2 scaffolds grown by innovative sputtering methods for high throughput hybrid photovoltaics. *Scientific Reports* **6**, 10.1038/srep39509 (2016). 10.1038/srep39509 PMC5175132 28000743

[33] Wang T (2013). Fabricating High Performance, DonorAcceptor Copolymer Solar Cells by Spray-Coating in Air. Advanced Energy Materials.

[34] Deegan RD (1997). Capillary flow as the cause of ring stains from dried liquid drops. Nature.

[35] Kavan L, Gratzel M (1995). Highly efficient semiconducting tio2 photoelectrodes prepared by aerosol pyrolysis. Electrochimica Acta.

[36] Mohamad D (2017). Optimized organometal halide perovskite solar cell fabrication through control of nanoparticle crystal patterning. Journal of Materials Chemistry C.

[37] Cotella G (2017). One-step deposition by slot-die coating of mixed lead halide perovskite for photovoltaic applications. Solar Energy Materials and Solar Cells.

[38] Fanton X, Cazabat AM (1998). Spreading and instabilities induced by a solutal Marangoni effect. Langmuir.

[39] Girotto C, Moia D, Rand BP, Heremans P (2011). High-Performance Organic Solar Cells with Spray-Coated Hole-Transport and Active Layers. Advanced Functional Materials.

[40] Griffin, J., Ryan, A. J. & Lidzey, D. G. Solution modification of PEDOT:PSS inks for ultrasonic spray coating. *Organic Electronics*, doi:10.1016/j.orgel.2016.11.011.

[41] Birnie DP (2013). A Model for Drying Control Cosolvent Selection for Spin-Coating Uniformity: The Thin Film Limit. Langmuir.

[42] Haas DE, Birnie DP (2002). Evaluation of thermocapillary driving forces in the development of striations during the spin coating process. Journal of Materials Science.

[43] Yi C, Li X, Luo J, Zakeeruddin SM, Graetzel M (2016). Perovskite Photovoltaics with Outstanding Performance Produced by Chemical Conversion of Bilayer Mesostructured Lead Halide/TiO2 Films. Advanced Materials.

[44] Gwyddion – Free SPM (AFM, SNOM/NSOM, STM, MFM, …) data analysis software (http://gwyddion.net/, 2016).

[45] Heo JH, Song DH, Im SH (2014). Planar CH3NH3PbBr3 Hybrid Solar Cells with 10.4% Power Conversion Efficiency, Fabricated by Controlled Crystallization in the Spin-Coating Process. Advanced Materials.

[46] Nelson, J. *The Physics of Solar Cells*. (Imperial College Press, 2003).

