# Supplementary material for: Spray-cast multilayer perovskite solar cells with an active-area of 1.5 cm2
Source: Sci Rep. 2017 Aug 11;7:7962. doi: 10.1038/s41598-017-08642-2 (PMC5554192; doi:10.1038/s41598-017-08642-2)
Supplement: Supplementary file 1 — Supplementary Information [file 41598_2017_8642_MOESM1_ESM.pdf]

Spray-cast multilayer perovskite solar cells with an active-area of 1.5 cm^2

James E. Bishop[1] and David K. Mohamad[1], Michael Wong-Stringer[1],

Alex Smith[2] and David G. Lidzey[1]*

1) Department of Physics & Astronomy, University of Sheffield, Hicks Building, Hounsfield Road, Sheffield, S3 7RH, U.K.

2) CREST, Wolfson School, Loughborough University, Loughborough, Leicestershire, LE11 3TU

*Corresponding author, email d.g.lidzey@sheffield.ac.uk

Supplementary Information

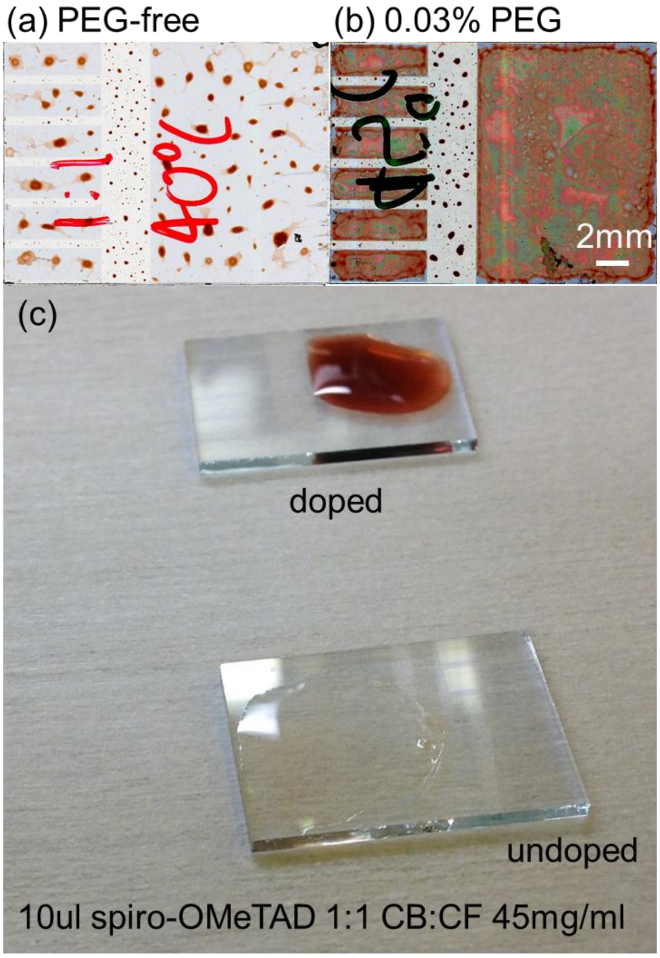

Figure S1 – Spray-coating spiro-OMeTAD ink onto an ITO/glass surface (a) without and (b) with 0.03 mg/mL PEG added to the ink. The increased contact angle can be seen in the image shown in part (c) when dopants are added to the ink.

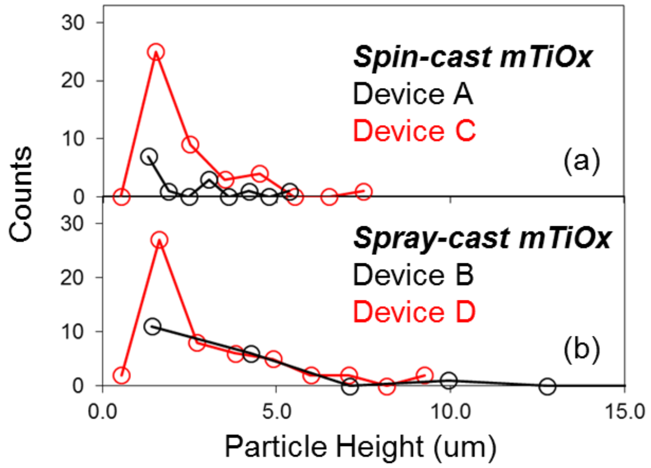

Figure S2 - The results of particle size analysis from topographic maps shown in Figure 4 are shown in parts (a) and (b): a particle height histogram from device A and C in shown in part (a) and device B and D in part (b)

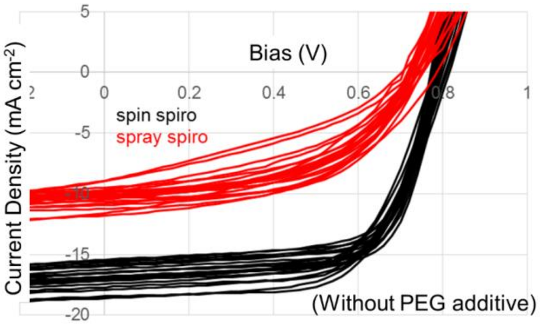

Figure S3 – Analogous spin-cast PSCs fabricated with spin-cast (black lines) and spray-cast (red lines) spiro-OMeTAD thin-films without the PEG additive.

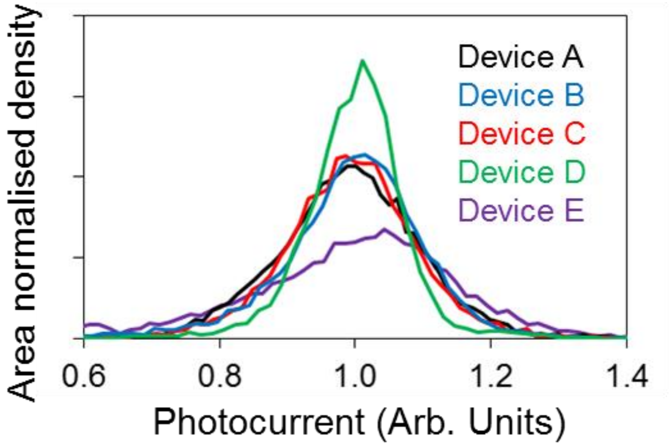

Figure S4 – Area normalised photocurrent histogram plotted from LBIC map data shown in Figure 4(f-g).

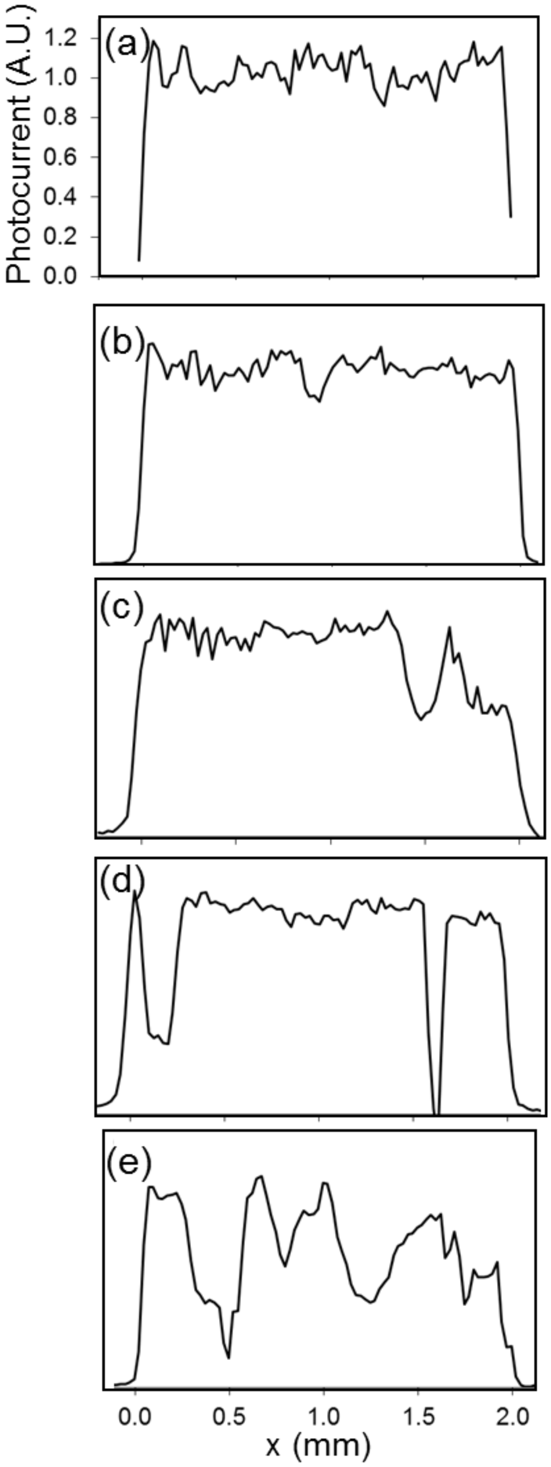

Figure S5 – Cross-sectional data from LBIC maps of Device A-E shown in Figure 4 (main text) shown in parts (a) to (e).

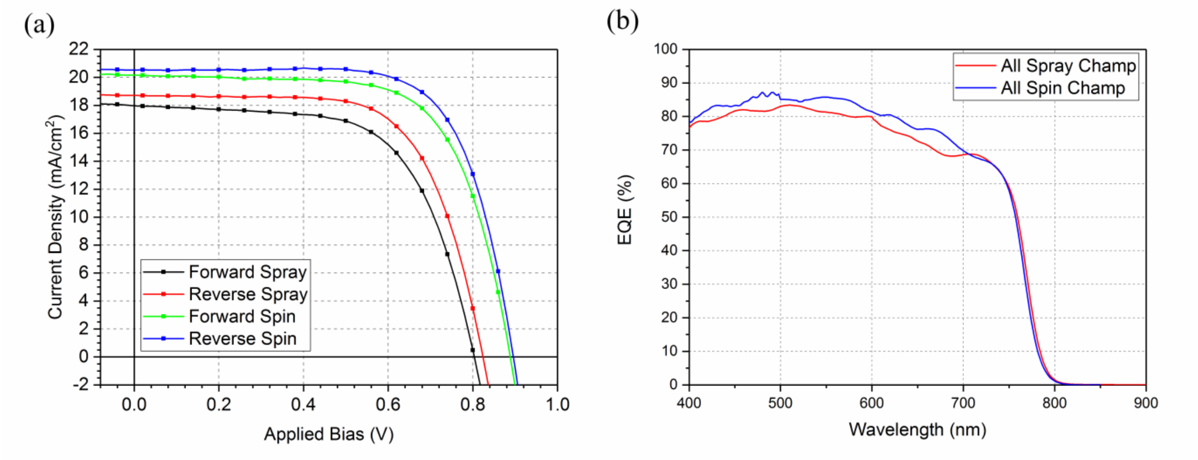

Figure S6 – Champion J-V [part (a)] from small-area device's E (all-spray-cast) and A (all-spin cast). The champion all-spin device has an efficiency of 12.9 % whilst the champion all-spray device has an efficiency of 10.2 %. Part b shows EQE spectrum's for these devices with respect to AM 1.5 illumination. We calculate the expected values for the J_{SC} as 18.4 mA cm^{-2} for device E and 19.0 for device A.

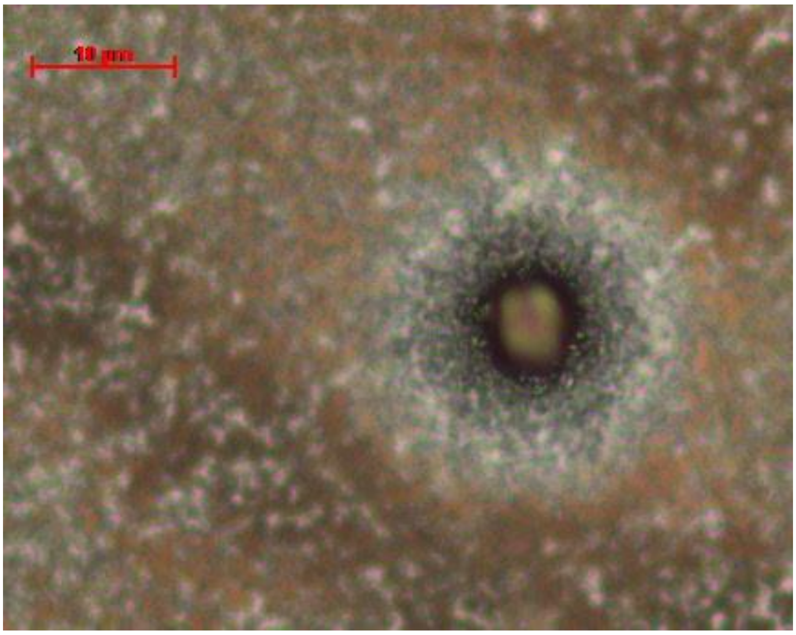

Figure S7 – Optical microscope image of aggregate in spray-cast perovskite film. The scale bar is 10 μm.

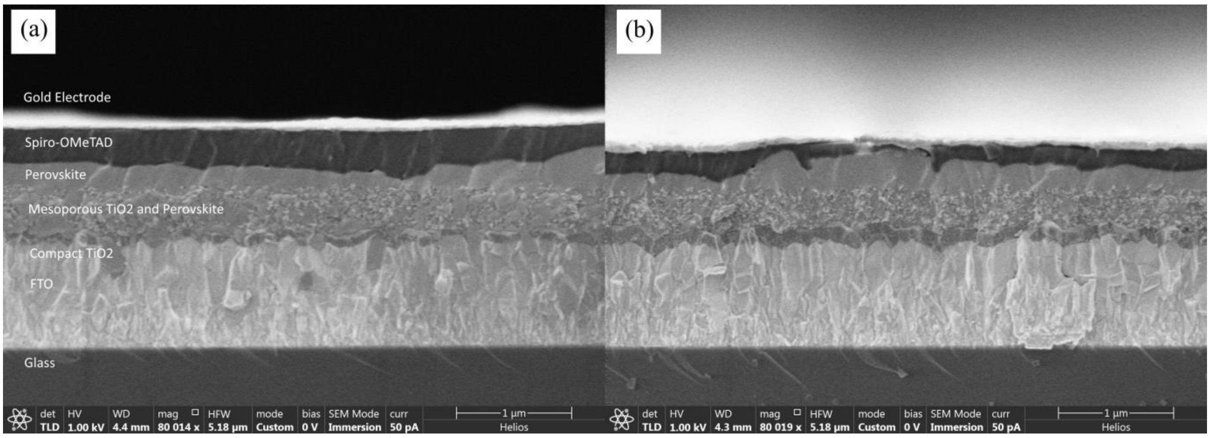

Figure S8 – Cross sectional SEM for device A (part a) and device E (part b).

IV-Curve Calibration Certificate

Issued by: CREST Photovoltaic Measurement and Calibration Laboratory | Page: 2 of 2

Report No.: 201611001

UKAS Accredited Calibration Laboratory No. 9171

Performance corrected to given test conditions				
Parameter	Value	U (k=2)	Unit	U (k=2)
Short Circuit Current (I_{SC})	17.90	±0.37	[mA]	2.04 %
Open Circuit Voltage (V_{OC})	847.65	±2.97	[mV]	0.35 %
Fill Factor (FF)	43.75	±0.22	[%]	0.50 %
Maximum Power Output (P_{MP})	6.64	±0.14	[mW]	2.15 %
Cell Efficiency (Eff)	6.59	±0.16	[%]	2.48 %

IV Curve:

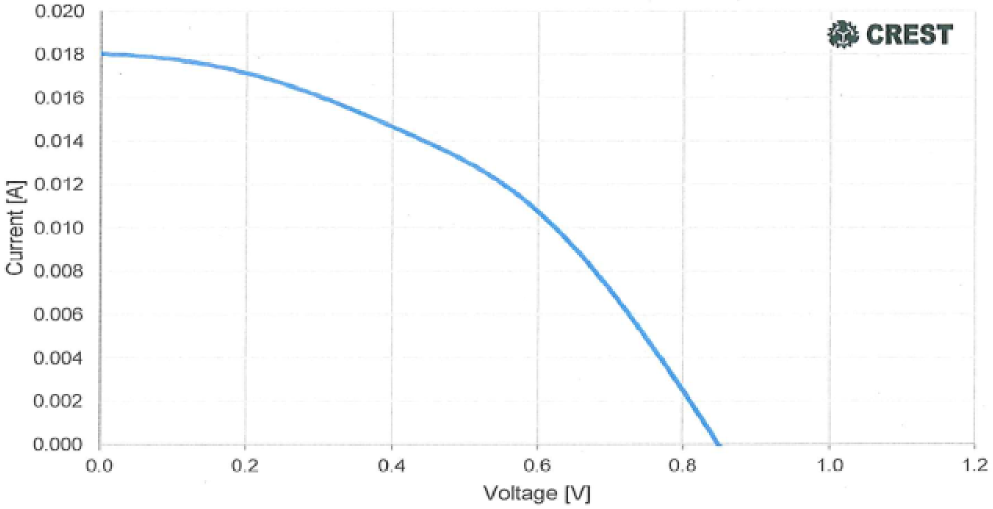

Figure S9- Extract from Calibration Certificate showing the results from independent JV measurements performed at CREST (University of Loughborough) on a large-area spray-cast device.
